# A Proposed Middle-Range Theory of Nursing in Hypertension Care

**DOI:** 10.1155/2018/2858253

**Published:** 2018-02-22

**Authors:** Eva Drevenhorn

**Affiliations:** Department of Health Sciences, Faculty of Medicine, Lund University, Lund, Sweden

## Abstract

Nursing in hypertension care comprises counselling about lifestyle changes, blood pressure measurement, and being a translator for the physician. For the patient, changing lifestyle means performing self-care. As not much in the form of research and guidelines for nurses is available, a middle-range theory of nursing in hypertension care was developed to guide nurses in their practice, in order to improve the nursing of patients and design studies for investigating nursing in hypertension care. Concepts are presented related to the patient (attitude and beliefs regarding health and sickness, autonomy, personality and traits, level of perceived vulnerability, hardiness, sense of coherence, locus of control, self-efficacy, and access to social support and network) and the nursing (applying theories and models for behavioural change in the consultation and using counselling skills, patient advocacy, empowerment, professional knowledge and health education, and supporting the patient). Then the concepts related to the consultation (communication, shared decision-making, concordance, coping, adherence, and self-care) are integrated with Orem's theory of nursing. Clinical and research implications of the theory are discussed.

## 1. Introduction

Nursing in hypertension care has been shown to comprise counselling about lifestyle changes, blood pressure measurement, and being a translator for the physician [1]. A more detailed description of the nursing interventions is presented by Hong [2]. When a nurse is a member of a team with other healthcare professionals in the care of the hypertensive patient, a reduction in blood pressure is seen [1]. This is a result of changed lifestyle, more correct intake of medication, and more frequent returns for follow-up visits. In this context for the patient, changing lifestyle and taking medicines mean performing self-care.

Patients with hypertension during pregnancy or other severe diseases are usually taken care of within specialised care, but adult patients with hypertension are mostly managed in primary care. Preferably the care is team-based so that the patients are met in a congruent way. The team can include, besides the nurse and physician, a physiotherapist or other professionals. The members of the team have to be aware that not many patients can identify any specific symptoms that are obviously connected with hypertension [3]; elevated blood pressure is most often detected when a patient is treated for some other ailment. The finding might surprise the patients, and then being faced with demands to perform self-care to adjust some figures on paper might be perceived as a real challenge. The nurse who sees the patient at visits to the clinic in primary care is presumed to have a health-promotional, holistic, and psychosocial approach in helping the patients to achieve blood pressure control.

Although nurses all around the world are involved in treating hypertensive patients, not many theoretical guidelines for nurses are available. There are theories of self-care and self-management of chronic illness in general, but not for hypertension specifically. A literature search found a theoretical framework for studying medication compliance in Chinese immigrants [4]. Otherwise the only papers found, with theoretical aspects, were a study that aimed to determine the effectiveness of a nurse's caring relationship according to Watson's Caring Model of blood pressure and quality of life [5], one that evaluated Orem's nursing self-care theory in hypertensive women (in Portuguese) [6], and another one that presented the middle-range theory of Attentively Embracing Story for practice implications with a client who had hypertension [7]. As these theoretical frameworks were not applied to embrace all aspects of nursing in hypertension care, a middle-range theory of nursing in hypertension care was judged to be needed to guide nurses in their practice, developing the nursing of patients and designing studies to investigate nursing in hypertension care. Former work in the development of the proposed theory has been presented [8, 9].

## 2. Methodology

**Figure 1:**
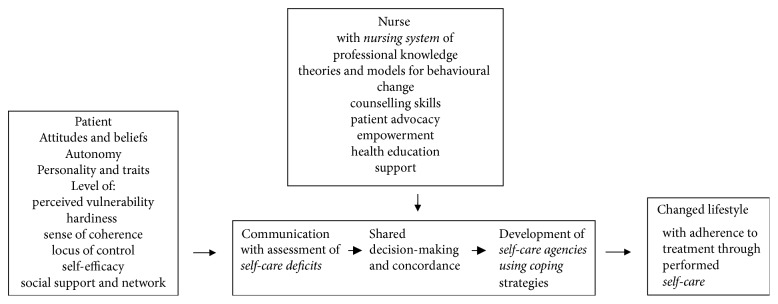
The constructed middle-range theory for nursing management of patients in hypertension care with the concepts involved in counselling about lifestyle changes and Orem's self-care deficit theory of nursing applied (shown in italics).

A middle-range theory is to be developed inductively from research and practice [10] and can be combined with existing theories [11]. Theories deriving from other disciplines and practice research can also be integrated. With this background, a middle-range theory of nursing in hypertension care is proposed (Figure 1) with concepts related to the patient, nursing in hypertension care, the encounter between the patient and the nurse, the expected outcomes of this encounter, and the integration of an existing grand theory of nursing.

In the development process of the proposed theory, extensive literature search and review were conducted to find concepts that mirrored counselling on lifestyle change and taking medicines to help the patient to perform self-care. Measuring blood pressure was omitted as this is to be done in a standardised way, which is well defined in hypertension guidelines. As for nursing in general, pieces from other disciplines such as medicine, sociology, and psychology fit in well; the databases PubMed, Cinahl, PsycINFO, SocSci, and Eric were used in the literature search along with the Cochrane Library in 2001, 2005, and 2014 (Table 1). The great output from the initial searches in 2001 was diminished through a thorough review to grasp what might be relevant for nursing in hypertension care. To find any relevant paper which could give any new aspects to be taken into account when elucidating what might be of concern for the patient and nurse in hypertension care, a variety of keywords (both MeSH terms and free text keywords) were used: adaptation, attitude, communication, compliance, coping, counselling, educational models, emotions, empowerment, health behaviour, health education, health promotion, hypertension, lifestyle, motivation, nurse-patient relations, nursing, patient self-determination, self-care, self-efficacy, and social support. The words were used separately as well as combined in the same structured way in every database. The procedure was documented to make it possible to repeat or refine the literature search.

During the review reference lists were also used to find cited original literature. While reviewing the findings from papers, books, and dissertations, a drawing was made to get an overview of relationships between the concepts and the way or order they fit in. Gradually the proposed model was formed with the concepts relevant for the patient and nurse arranged to form the model (Figure 1). The background for choosing keywords consisted of studies on nursing in hypertension care for several years while obtaining bachelors', masters', and doctoral degrees. Moreover, personal experience as an advanced nurse in primary care running a nurse-led hypertension clinic was crucial. The theory was first presented as a part of the framework of the author's thesis and some years later it was decided to rewrite the framework into a paper to be diffused among colleagues for discussion and review. Some of the concepts included are well defined but others are less well developed and defined and also not used in research with patients with hypertension. The proposed theory is work in progress and further development is needed. The theory is presented on these assumptions.

## 3. Definitions of Concepts

In the paper research results from each concept are presented whenever any study with hypertensive patients involved has been found regardless of the study's age. First, concepts related to the patient are presented followed by the concepts related to nursing in hypertension care, concepts related to the consultation where the patient and nurse meet, the integration with Orem's self-care deficit theory, and finally the expected outcome (Figure 1).

### 3.1. Concepts Related to the Patient

Concepts that are related to the patients' ability, disposition, and willingness to change lifestyle and take medicines are presented here. Factors comprising attitude and beliefs regarding health and sickness, autonomy, personality and traits and level of perceived vulnerability, hardiness, sense of coherence, locus of control, self-efficacy, and access to social support and network are of importance.

#### 3.1.1. Attitude and Beliefs regarding Health and Sickness

An attitude is a psychological tendency that is expressed by evaluating a particular entity with some degree of favour or disfavour [12] and a value can be defined as a personal belief about the worth, desirability, goodness, truth, and beauty of a particular idea or object [13]. One's concept of health is tied into one's belief systems, which means that no patient will follow instructions that the person does not believe will work or will work towards a goal not valued. For that reason the patients' values can be considered the basis for decision-making on whether to perform lifestyle change or not. Patients hold ideas about whether hypertension is a disease or not, whether the drug is necessary evil or a help, and how the drug should be taken and its effects [14], and their perceptions of the cause of hypertension, experiences of symptoms, and beliefs about the treatment can influence their self-management [15]. Assessing these attitudes and beliefs is an important first step to develop a plan of care for the patient [16].

#### 3.1.2. Autonomy

Autonomy is defined as self-government; that is, people are autonomous to the extent to which they are able to control their own lives [17]. The hypertensive patient is facing demands to make lifestyle changes that are to be lifelong and for that reason the decision has to be autonomously made [18]; otherwise the maintenance of the new behaviour is likely to decrease. It is therefore crucial that nurses can identify that the patient is sufficiently autonomous, which means that the patient can understand and retain the information relevant to the decision in question, believe the information, and weigh up the information to arrive at a decision.

#### 3.1.3. Personality and Traits

A personality is a blend of two or more traits [19], which are factors that determine our conduct in many different situations and also when it comes to changing lifestyle. Traits are divided into the dimensions extraversion (active, assertive, enthusiastic), agreeableness (appreciative, forgiving, generous), conscientiousness (efficient, organised, responsible), neuroticism (anxious, tense, worrying), and openness (artistic, imaginative, insightful). States or moods are singular occurrences. Older adults, >65 years of age, who score high in conscientiousness are shown to perceive themselves as having health competence, which in turn was strongly related to health behaviours (exercise, dietary/health information, and relaxation/social support) [20]. Scoring high in conscientiousness might also promote self-control skills that can affect adherence to treatment [21]. Type A behaviour pattern, aggressively seeking to achieve more and more in less and less time, appears to increase blood pressure reactivity to external stressors in persons with mild hypertension [22].

#### 3.1.4. Vulnerability

Vulnerability is defined in nursing literature as an externally evaluated risk (by a person outside the experience) or as an experiential state (as understood by the person herself) [23]. The individual's perceptions of self and challenges to self and of resources to withstand such challenges create the individual person's perceived vulnerability. The degree of vulnerability changes with the degree of environmental support and personal resources [24]. Psychological effects of vulnerability could give, among other things, a feeling of not belonging, helplessness, fear, anger, uncertainty, loss of control, isolation, anxiety/worry, and powerlessness. Likelihood to engage in positive action to change behaviour depends on whether the individual perceives himself as being well, chronically ill, or having an acute illness [25]. It is concluded that a hypertensive person can be placed between well and chronically ill, which means that motivation to learn about any aspect of healthcare is based upon the acceptance of self-responsibility for health. The person does not feel vulnerable or at any risk.

#### 3.1.5. Hardiness

The concept of hardiness was first introduced as a personality characteristic with the dimensions of commitment (active involvement in one's life activities), control (belief in the ability to influence the course of one's life events), and challenge (change is normal and growth-producing) [26]. Hardy individuals have a higher sense of commitment to self and work, perceive life change as challenging rather than threatening, and maintain a sense of control in life rather than powerlessness. Hardy persons in hypertension and RA groups are reported to be more likely to participate in patient education programmes, and this was related to better physiological functioning [27].

#### 3.1.6. Sense of Coherence

The theory of sense of coherence is a salutogenetic model (salute = health, genesis = origin) [28]. A person with generalised resistance-resources such as money, good self-strength, cultural stability, and social support can manage to make stressors understandable, that is, have a feeling of coherence. This also involves coping strategies, that is, a plan for behaviour, social support, and cultural factors. A person with a feeling of coherence has an attitude that expresses a long-lasting and deep faith in the world's predictability and thinks there is a high likelihood that things will turn out as well as you can reasonably expect. It does not imply that one is in control but that one is involved as a participant in the processes shaping one's destiny as well as one's daily experience. It is not important whether power is in our hands or elsewhere but that the location of power is where it is legitimately supposed to be.

#### 3.1.7. Locus of Control

As psychosocial dynamics influence health behaviour, Rotter et al. [29] developed the idea that generalised expectancies and reinforcement make a person create a sense of locus of control over the events in his life. If the effect of reinforcement is perceived as not being entirely derived from a person's own actions but the result of luck, chance, or fate, as under the control of powerful others, it is labelled a belief in external control. If the person perceives that the event originated from his own behaviour or his own characteristics, it is termed a belief in internal control. It is reported that patients with hypertension who scored high on internal control adhered to their medication had a controlled blood pressure [30].

#### 3.1.8. Self-Efficacy

Perceived self-efficacy refers to beliefs in one's capabilities to organise and execute the courses of actions required to produce certain practical attainments [31]. If people believe they have no power to produce results, they will not attempt to make things happen [32]. Perceived self-efficacy is a uniformly good predictor for diverse forms of behaviour. Efficacy beliefs affect performance both directly and by influencing intentions. Suggested areas for research include nonpharmacological treatment for hypertension, adherence to diet and medication regimens for lipid control, and interventions to improve exercise habits [33]. Effects of health literacy on self-efficacy and knowledge about health matters are also a factor to consider [34].

#### 3.1.9. Social Support and Network

There is no universally accepted definition or conceptualisation of social support, but Lindsey [35] defines social support as provision of information that leads people to believe they are cared for, loved, esteemed, valued, and a member of a network of communication and mutual obligation. There are four types of social support: informational (provision of information that the person can use in coping with personal and environmental problems), appraisal (transmission of information relevant to self-evaluation), instrumental (access of the individual to behaviours that directly help in time of need), and emotional (provision of empathy and demonstration of love, trust, and caring). Social network that provides social support is defined as a group of people with whom the person has social connections, which may be formal or informal and can be described by size, density, and complexity. In studies high social network score is associated with lower systolic and diastolic blood pressure for both sexes [36]. In China it was found that social support, education, and duration of diagnosis of hypertension were significant predictors of treatment adherence [37].

### 3.2. Concepts Related to Nursing in Hypertension Care

Concepts of importance for the nurse when nursing patients in hypertension care are presented here. They involve applying theories and models for behavioural change in the consultation and using counselling skills, patient advocacy, empowerment, professional knowledge and health education, and supporting the patient.

#### 3.2.1. Theories and Models for Behavioural Change

There are several theories and models developed to understand what determines behaviours. In healthcare the health belief model [38] is the most frequently used model in behaviour change. In this model goal-setting, decision-making, and social learning are integrated for making one's own decisions depending on positive or negative attitudes. Perceived susceptibility and barriers for behaviour change are included too. Other models that are useful for nurses in treating patients regarding lifestyle changes are, for example, the transtheoretical model (TTM) or stages of change model (SOC) [39] where the learner goes through a cycle from precontemplation to contemplation, preparation, and action to maintenance of new behaviour; the self-regulatory model (SRM) [40] where motivation for changing lifestyle is dependent on perceived threat; the protection motivation theory (PMT) [41] according to which judging the severity of health threats affects coping responses; or the health promotion model (HPM) [42] where perceived benefits, barriers to action, self-efficacy, interpersonal and situational influences affect behaviour change. In our own studies it was shown that nurses educated in the SOC model may have some help when using the model in counselling hypertensive patients [43].

#### 3.2.2. Counselling Skills

The process of counselling can be defined as “the means by which one person helps another to clarify his life situation and to decide further lines of action” (p. 2) [44]. To act, the patient needs to be able to identify those things she has to do, stop doing, continue to do, and accept. Morrison and Burnard [45] suggest definitions of counselling as helping people to come to terms with a problem, enable a person to find solutions to problems, or help people to help themselves. Motivational interviewing (MI) [46] is a patient-centred goal-oriented counselling style for addressing the coercion of ambivalence about change. Using this counselling style is a way to preserve patient-centredness, empathy, and patient autonomy in the consultation. Own studies show that nurses after consultation training fulfilled more aspects of patient-centredness after the training [47] and were more focused and discussed lifestyle factors to a greater extent with their patients [48]. Furthermore effects on patients' weight parameters, physical activity, perceived stress, and the proportion of patients who achieved blood pressure control have been demonstrated [49].

#### 3.2.3. Patient Advocacy

An advocate is “one who pleads the cause of another” (p. 439) [50]. The concept of advocacy is linked with concepts of morality, ethics, autonomy, and patient empowerment. Gadow [51] proposes that the concept of advocacy is the philosophical foundation and ideal of nursing. The nurse is uniquely suited for fundamental and existential advocacy distinct from just providing the patient with any correct and objective information and being paternalistic. Schwartz [52] states that the advocate should inform the patient and promote informed consent, empower the patient and protect autonomy, protect the rights and interests of the patient, ensure access to available resources, support the patients, and represent the views/desires of the patients and not just their needs.

#### 3.2.4. Empowerment

The word empower means “to authorise, to license, to impart power, to enable, and to permit” and a definition of the concept of empowerment could be “in a helping partnership it is a process of enabling people to choose to take control over and make decisions about their lives” (p. 309) [53]. Rappaport [54] views empowerment as the vehicle by which problems in living may be handled, and it suggests a sense of control over one's life in personality, cognition, and motivation. The empowerment approach to health education seeks to increase patient autonomy and expand freedom of choice [55]. The nurse can contribute by doing assessments of patients' psychosocial health and appraisal of health and health risks. Personal empowerment is promoted by encouraging people to identify their values, needs, goals, and the resources they have to solve problems. Awareness, freedom, choice, and responsibility are the four pillars of empowerment. Nurses' counselling was found to contain both empowerment and nurse-centred features in a study in hypertension care, which alternated during the conversations but nurse-centred features were predominant [56].

#### 3.2.5. Professional Knowledge

To be able to perform all the tasks that are included in nursing in hypertension care, the nurse has to be updated in the latest guidelines for hypertension treatment, both pharmacological and nonpharmacological. Being the leader of the team [1] around the patient, as often is the case, the nurse has to have knowledge about how to value the results of the patient's blood tests regarding, for example, blood lipids and also body measurements such as waist circumference and body mass index. The nurse also needs to be able to estimate the patient's individual risk profile.

#### 3.2.6. Health Education

The importance of health teaching as a part of nursing has been recognised for a long time. A professional model for teaching in nursing practice consists of four components: social service ideal (characteristics of the profession), practice environment (environment that influences the practice), client state (nurse's view of the client), and nursing practice strategies (unique nursing interventions) [57]. This model is applicable in nursing in hypertension care as it encompasses a professional autonomy and spirit which the nurse must master as she is often managing the clinic on her own. She must also have understanding of man's physiological and psychosocial state to make an assessment of the patient to determine the nature of teaching needed. This encompasses a holistic view. The teaching can be performed individually or in group. Hypertensive patients were interviewed about their views on a working booklet used in consultations at nurse-led clinics where the nurses had received counselling training [58]. The booklet was reported to have been read several times by some patients, but a few patients did not remember receiving it. Individual health education in primary care is reported to give lower systolic and diastolic blood pressure and body mass index and improve self-efficacy regarding medication adherence [59].

#### 3.2.7. Support

The patient often gets social support from, for example, their next of kin and friends, but the nurse also has to support the patient regarding the specific individual lifestyle problems the patient has. Support and MI skills can also be needed to help the patients to increase their self-efficacy to succeed in changing lifestyle [46]. This could also be expressed as interpersonal transactions that include the expression of positive affect of one person towards another, the affirmation of another's behaviours, perceptions, or expressed views, and the giving of symbolic or material aid to another person [35, 60].

### 3.3. Concepts Related to the Consultation and the Expected Outcome

In the consultation the patient and the nurse meet. Presented below are specific concepts related to this meeting and the expected outcome, where the nurse is communicating with the patient to reach a shared decision in concordance with the patient regarding the self-care the patient should perform. To be able to perform self-care the patient uses different coping strategies to adhere to the decided treatment.

#### 3.3.1. Communication

Caring is an interpersonal process and builds the rapport between nurse and patient. A conceptual framework has been developed for classifying varieties of interpersonal intervention between nurse and patient [45]. The authoritative interventions are prescriptive (to offer advice, make suggestions), informative (to offer information), and confronting (to challenge) and the facilitative ones are cathartic (to enable the release of emotion), catalytic (to “draw out”), and supportive (to encourage or validate). An interpersonally skilled person is one who can move appropriately and freely between the various categories as a means of guiding therapeutic action. All of these interventions can be found in MI [46] except the authoritative kind, which is seen as counterproductive for creating rapport with the patient. A client-centred approach means that the client himself is best able to decide how to find solutions to his problems in living [61]. All the skills of communication are also skills of counselling as listening, paraphrasing, challenging, and goal-setting [62]. Communication can be verbal or nonverbal. Interviewed hypertensive patients reported that the nurse listened and they had been guided and motivated to perform lifestyle changes after the nurses' counselling training [58]. There were more informed thoughts about how to manage lifestyle in this group compared to the control group.

#### 3.3.2. Shared Decision-Making and Concordance

The Swedish Health and Medical Services Act [63] states that care should be given based on respect for patients' own decisions and their integrity. Toop from New Zealand states in a paper [64] that a patient-centred approach based on mutual participation has gained increasing support, and in the US the concept of sustained partnership between patient and clinician has been included in a definition of primary care. Mutual goal-setting is a process whereby nurse and patient collaboratively define a set of patient goals and agree on the goals to be attained. Goal-setting is an essential part of problem solving and the nursing process. Charles et al. [65] argue that the shared treatment decision-making model has four necessary characteristics: both the physician and patient are involved in the treatment decision-making process, they share information with each other, they take steps to participate in the decision-making process by expressing treatment preferences, and a treatment decision is made. Charles and contributors focus on physicians' interaction with patients but here the principles for decision-making are generally applicable.

Through the concordance website managed by the RPSGB concordance coordinating group the new concept will begin to grow [66]. The historical background was the problem of noncompliance with medication and growing knowledge about the beliefs that people hold about their medication and about medicines in general. These beliefs decide whether the individual will comply with a prescription or not. The concept can also be extended to other treatment modalities such as behaviour change. In the consultation concordance should be based on a negotiation between equals. Concordance implies the approach of bringing patients into a full therapeutic partnership, that a patient's decision-making preferences may change with time and circumstances, and that if the patient has relatively more authority or control in the consultation, the prescriber will have less. Moreover, the patient might choose a treatment other than that proposed by the prescriber and this is not a failure but a success of care, and if this happens, it is no basis for rejection of the patient. In MI the word concordance is not used, but the idea of the MI spirit means that the patients are viewed as valued partners whom the counsellor wants to help to make his or her own decisions regarding health matters [46]. Shared decision-making and concordance do not overlap but rather supplement each other.

#### 3.3.3. Coping

Coping comprises the person's strategies to handle trying situations and demands that are appraised as taxing or exceeding the resources of the person [67], such as being diagnosed as having hypertension. A person can use strategies to handle the stress through acceptance, tolerance, medication, avoidance, and so forth or strategies to change the situation that caused the stress [68]. A vigilant coping strategy is directed to the problem in an effort to prevent or control it [67] and could mean information searching or systematic problem solving. Coping by avoidance could be jogging, relaxation, vacation, hobbies, wishful thinking, eating, drinking, smoking, using drugs or medications, or sleeping. Arora and McHorney [69] used questionnaires to study whether 2472 chronically ill (hypertension, diabetes, congestive heart failure, myocardial infarction, depression) persons preferred an active or passive role in medical decision-making. They found that patients using an active coping strategy preferred an active role in meetings with health professionals.

#### 3.3.4. Adherence

There has been a shift through the years from using the term compliance to using adherence, though many authors still use the word compliance. Compliance has authoritative connotations, implying that the practitioner expects the patient to follow rules that are for the patient's own good [13] or that the patient is a passive responder to the clinician's authoritative demands [70]. Noncompliance means that the patient asserts the right of self-responsibility. Adherence can be defined as “the extent to which a person's behaviour (in terms of taking medications, following diets, or executing lifestyle changes) coincides with medical or health advice” according to Haynes in 1979 [71]. Expectancy of internal control over health and hypertension and knowledge of the treatment regimen are significant determinants of adherence. Poor adherence to treatment can also mean that the person actually has not changed his opinion, has not understood the message completely, is not convinced about having to change behaviour to avoid illness, or has not received any help to adopt other habits [72]. Sensitivity of symptoms is generally an indicator of disease and used as a motivator and guide for treatment. Hypertension is a good example of poor sensitivity and therefore a bad motivator for treatment adaptation [40]. Regarding adherence to medication Allen [73] states that the hypertensive patient feels well and there will be no increase in perceived well-being when medication is taken, which could contribute to noncompliance. The cost of the medication [74], especially among older people [75], is an important barrier to adherence.

#### 3.3.5. Self-Care

Self-care can be defined as activities initiated or performed by an individual, family, or community to achieve, maintain, or promote maximum health [42]. Within the medical model, self-care has been defined as self-care in illness, compliance with therapeutic regimens, and active participation in rehabilitative activities. Self-care for health promotion requires that “clients have the knowledge and competencies that can be used to maintain and enhance health” (p. 98). A similar definition is used by Levin [76] meaning that self-care is a process where a lay person can function effectively in taking care of his or her own health promotion and prevention and disease detection and treatment. Within nursing the concept of self-management seems to encompass not only different coping strategies but also health-promotive actions, interaction with healthcare providers, adherence to treatment, monitoring health status, making care decisions, and management of the impact of the illness on health [77].

### 3.4. Integration with Existing Theory of Nursing

All the defined concepts related to nursing in hypertension care form the basis for nursing interventions, and the nurse makes an assessment of the patient's self-care demands in communicating with the patient to help the patient to change lifestyle, that is, to perform self-care. For that reason Orem's self-care deficit theory of nursing [78] is integrated as a natural ingredient in the proposed middle-range theory.

The self-care deficit theory of nursing consists of three parts: theory of self-care, theory of self-care deficit, and theory of nursing systems [78]. Presuppositions for the theory are that people develop and exercise intellectual and practical skills through learning and manage themselves to sustain motivation essential for continuing daily care of themselves. Self-care is an action of persons who have developed the capabilities to take care of themselves in their environmental situations. They have the agency or power to act to regulate internal and external factors that affect their own functioning and development, that is, in the interest of life, health, and well-being. The ability to perform self-care, the self-care agency as a specific power of individuals, differs depending on capabilities and circumstances related to the self-care demands and health disorders. Self-care deficit arises when capabilities for self-care, because of existing limitations, are not equal to meeting some or all of the components of their therapeutic self-care demands. In hypertension care the self-care deficit could mean that the patient has a lack of knowledge about or is not motivated to perform lifestyle changes or to start taking medicines. These deficiencies mean that the patient cannot develop appropriate self-care agency.

To meet the patient's self-care deficit, nurses produce systems of nursing actions, nursing agencies [78]. In hypertension care these agencies most often are supportive-educative to help the patient to regain his self-care. The supportive-educative system is the only system where a patient's requirements for help are confined to decision-making, behaviour control, and acquiring knowledge and skills. The nursing actions could be of long or short duration.

The concepts related to nursing (theories and models for behavioural change, counselling skills, patient advocacy, empowerment, professional knowledge, health education and support) form the nursing system in hypertension care which the nurse brings into the encounter with the patient. The patients in turn bring certain attitudes and beliefs, a view of health, need for autonomy, their own personality and traits, perceived vulnerability, hardiness, a sense of coherence, locus of control, self-efficacy, and social support and network into the encounter. All these factors affect behaviour and thus the patients' habits. In the interaction the nurse uses appropriate parts of the nursing system to assess the patient's self-care deficits. From the interaction in the patient-centred communication, shared decision-making should emerge, with goal-setting in concordance between the patient and the nurse. From the decisions made about performing self-care the patient has to develop self-care agencies to perform behaviour change through his own coping and with assistance from nursing agencies, that is, the nurse's interventions. The desired outcome is changed lifestyle with the goal of adherence to treatment and maintenance of new behaviour. This was demonstrated in our own research in measuring hypertensive patients' exercise of self-care agency [79] where counselling training gave an increase in the patients' self-care agency scores, which was significantly correlated with increased physical activity and improved satisfaction with information about medication [80], which mirrors adherence to treatment. From Turkey it is similarly reported that hypertensive patients' educational level and social insurance situation affect the measured self-care agency score [81].

## 4. Clinical and Research Implications

Nursing in hypertension care has been criticised for not being fully professionally performed and for not having any structure for the counselling in the consultations [82]. Nurses could have a prominent position in the treatment of hypertensive patients, but to achieve this, the standard of the nursing needs to be enhanced. For that reason, a theory can be necessary to give a basis for the nurses to be able to develop guidelines for their own practice.

In being patient-centred in their communication, nurses must see the individual patient with his or her needs. It is then necessary for the nurse to have knowledge about what factors may affect the patient, factors that might help or be less helpful for the patient to manage to perform necessary lifestyle changes or take medicines. A patient can have the idea that living a healthy life is not for him as he is predestined because of a heavy heredity for cardiovascular diseases. His attitude is to be careless and his beliefs are that it is not worthwhile to attend to his weight. He maintains his stance to preserve his autonomy. He gives the impression of being conscientious as he is efficient and organised. Regarding his hypertension, he feels powerless (vulnerable) and thus has a low level of hardiness. He has also a low sense of coherence as he has low self-strength and feels that he is lucky if he will reach the age of fifty (external locus of control). However, he would score high on self-efficacy because when he decides to do things, he has a strong belief in being successful. He has also social support from his family and a social network that would be of help if he could be motivated to change lifestyle. This is exemplified in Table 2. In meeting patients, the experienced nurse presumably gets an impression of the individual patient and can report most of the statements listed in the scenario above, but the nurse would probably not be able to give a theoretical background and relate to these concepts. To be able to study and put words on what nursing in hypertension care means, a theoretical foundation is necessary.

To integrate Orem's self-care theory is particularly relevant to patient teaching with development of self-care skills [13, 32] as this is the goal for hypertensive patients and there is acceptance of the theory with its application to a variety of client populations and clinical settings (Whetstone and Reid, 1991). For many patients with hypertension it is hard to understand how the figures describing their blood pressure level are going to affect their lives. Most patients do not have symptoms that are easy to relate to the diagnosis. This means that their challenge is more demanding than those of many other patients, and it is also a big challenge for the nurse to help the patient to understand the seriousness of the figures and to motivate the patient to perform the lifestyle changes that are needed. For that reason a more detailed and applied theory is needed for the nurse in hypertension care than using Orem's grand theory alone or even a middle-range explanatory theory of self-management behaviour [83].

Intervention studies on nursing in hypertension care have been performed and show that consultation training can give nurses a structure for the consultations [48] and that nurses can be more patient-centred in their counselling [47]. Using behavioural models as a theoretical framework has also been found to be of use [43]. Other research shows that integrating behavioural models with counselling technique as in MI [84] is helpful for healthcare providers of different professions. In doing this the provider gets a structure for the consultation and can be more effective in treating the patients. For nurses the application of a theory in hypertension care can give even deeper understanding of the patients' challenges and also of their own professional nursing actions.

Further research could be directed to different aspects of the application of the theory to clinical settings or to the theoretical relationships between the concepts. In clinical settings it would be of interest to study nurses' views on applying the theory in their practice and also hypertensive patients' views of how they perceive their treatment and their experiences of performing self-care. Intervention studies could be performed where the nursing care is based on the theory to see if this makes any difference on patient outcomes. The relationships between the concepts and a development of the different concepts need attention too. There are several questions to be answered such as what the differences between concordance and shared decision-making are, whether it is important to incorporate levels of hardiness, sense of coherence, and locus of control in the theory, and whether it makes a difference using the word patient-centredness in defining counselling skills instead of person-centredness. According to Higgins and Moore's definitions [85], this proposed theory is predictive, that is, anticipating a particular set of outcomes, and has the components of identified and defined concepts, assumptions that clarify the basic underlying truths, a context within which the theory is placed but lacks identified relationships between and among the concepts. For that reason, views from the world of nurses interested in the development of theorising nursing in hypertension care are most valuable.

## Figures and Tables

**Table 1:** Searches made in the different data bases during the years. The searches in 2001 started from the year of the start of the respective database.

2001		2005		2014	
Database (covering years)	Number of relevant findings	Database (covering years)	Number of relevant new findings	Database (covering years)	Number of relevant new findings
PubMed (1966–2001)	520	PubMed (2002–2005)	15	PubMed (2006–2014)	4
Cinahl (1982–2001)	326	Cinahl (2002–2005)	11	Cinahl (2006–2014)	4
PsycINFO (1967–2001)	298	PsycINFO (2002–2005)	15	PsycINFO (2006–2014)	2
SocSci (1986–2001)	147	SocSci (2002–2005)	3	SocSci (2006–2014)	2
Eric (1966–2001)	0	Eric (2002–2005)	0	Eric (2006–2014)	0

**Table 2:** A schematic description of how the nurse could make use of the proposed theory in hypertension care. All counselling sessions with patients need not follow the same order, and one concept is not used just at one particular time; for example, communication and professional knowledge are used throughout the consultation. The treatment mentioned here is lifestyle change.

Process of the consultation	Concepts related to the meeting	Concepts related to the patient	Concepts related to the nurse
Creating rapport with the patient	Communication		

Who is this person?		Personality and traits	Counselling skills

Assessing the patient's individual risk profile and telling the patient the result	Assessment of the patient's self-care deficits		Professional knowledge

What has the patient to say?		Attitudes and beliefs regarding health and sickness Sense of coherence Perceived vulnerability Hardiness	

The patient has questions		Autonomy	Health education

Discussion of pros and cons of lifestyle change where the patient reaches a decision whether to make a change or not	Shared decision-making and concordance	Locus of control	Patient advocacy Theories and models for behavioural change

The patient is willing	Development of self-care agencies using coping strategies	Self-efficacy Social support and network	Empowerment Support

Follow-up	Adherence to changed lifestyle through performed self-care		
